# Do regular exercise, gender, and age influence smartphone addiction? Analyzing screen time and smartphone deprivation symptoms

**DOI:** 10.3389/fpsyg.2025.1586762

**Published:** 2025-05-08

**Authors:** Neha Pirwani, Angéla Somogyi, Attila Szabo

**Affiliations:** ^1^Doctoral School of Education, Faculty of Education and Psychology, ELTE Eötvös Loránd University, Budapest, Hungary; ^2^Institute of Health Promotion and Sport Sciences, ELTE Eötvös Loránd University, Budapest, Hungary; ^3^Faculty of Health and Sport Sciences, Széchenyi István University, Győr, Hungary

**Keywords:** behavioral addiction, biological sex, problematic smartphone use, physical activity, withdrawal symptoms

## Abstract

**Background:**

Headless smartphone use, known as smartphone addiction (SA), is associated with mental health issues and behavioral dependencies. While physical activity (PA) has been suggested as a protective factor, research remains inconclusive. The present study examines whether regular exercise influences SA, smartphone deprivation, and daily smartphone screen time while assessing gender and age as risk factors.

**Methods:**

This cross-sectional study with 701 participants (392 exercisers, 309 non-exercisers) assessed SA with the Smartphone Application-Based Addiction Scale (SABAS) and deprivation feelings with the Hungarian Smartphone Withdrawal Symptom Scale (HSWSS). Daily smartphone use was self-reported. Multivariate analysis of covariance examined the effects of exercise status, gender, and age on SA-related variables.

**Results:**

Exercise status did not significantly affect SA, deprivation feelings, or screen time (*p* > 0.05). However, the correlation between SA and smartphone screen time was stronger (*p* < 0.05) in non-exercisers (*r* = 0.407) than in exercisers (*r* = 0.274). Gender and age were significant predictors: females reported higher SA and usage, and younger age was associated with greater SA, deprivation symptoms, and more screen time.

**Conclusion:**

While exercise may not reduce SA, exercisers exhibit a weaker link between SA and smartphone use, suggesting less problematic usage, possibly due to more utilitarian (e.g., sports-related) rather than hedonic use. Females report higher SA, feelings of deprivation, and screen time than males, while age correlates with increased SA, feelings of deprivation, and screen time. Future research should examine psychosocial mechanisms and diverse smartphone usage patterns in addiction in connection with exercise behavior.

## Introduction

Smartphones have become an essential part of daily life. As of 2025, an estimated 7.49 billion people worldwide use smartphones (Statista, 2023), while the global population is projected to reach 8.09 billion (United States Census Bureau, 2025). These figures suggest that almost all inhabitants of the planet, excluding young children, own a device. The widespread adoption of smartphones is mainly attributable to their multifunctionality, serving as convenient tools for healthcare, communication, shopping, sports and exercise tracking, education, entertainment, etc. With increasing digitalization, smartphones have transitioned from luxury to necessary items (Lee et al., 2014). Smart-home systems, such as Alexa and Google Home, have further embedded these devices into daily routines. For individuals with disabilities, applications like ‘Augmentative and Alternative Communication for autism,’ ‘Lookout’ for visually impaired people, and ‘Google Live Transcribe’ for the hearing impaired provide essential support.

While smartphones offer numerous benefits, excessive and headless (uncontrolled and irresponsible) use can lead to problematic behaviors. Smartphone applications facilitate access to health-related information, social support, and well-being (Bert et al., 2014; Kang and Jung, 2014). However, loss of control over smartphone use can result in compulsive behaviors with adverse health effects, which are recognized as a hallmark of smartphone addiction (SA).

The term “smartphone addiction” is widely used in the literature (Khan and Khan, 2022). However, it more accurately refers to compulsive and maladaptive engagement with smartphone-based applications rather than dependence on the device (Pirwani and Szabo, 2024a). The present study defines SA as a predisposition toward maladaptive smartphone-based application use, following the addiction pathway model proposed by Billieux et al. (2015). It is characterized by excessive engagement, impaired self-regulation or control, and feelings of deprivation, such as irritability, restlessness, and anxiety when access is restricted.

Daily screen time is a key behavioral indicator of SA, particularly when usage is driven by non-utilitarian motivations such as social media and entertainment (hedonic) consumption (Csibi et al., 2017). Studies have consistently shown that excessive smartphone screen time is associated with difficulties in self-regulation, mirroring behavioral addiction patterns. Several psychometric tools, including the Smartphone Application-Based Addiction Scale (SABAS) and the Brief Addiction to Smartphone Scale (BASS), incorporate both excessive screen time and withdrawal symptoms in their assessments, underscoring the importance of jointly evaluating these factors. The present study aims to better understand problematic smartphone behavior by assessing withdrawal symptoms, daily screen time, and regular exercise status, which has been associated with lower SA (Pirwani and Szabo, 2024b).

Excessive smartphone screen time is connected to adverse health outcomes, including reduced physical activity (PA; Olson et al., 2022). Smartphone addiction shares commonalities with other behavioral addictions like technological addiction, internet gaming disorder, and gambling disorder (Lin et al., 2017). Individuals exhibiting SA symptoms often experience functional impairment, reduced productivity, and withdrawal symptoms, as well as adverse psychological outcomes such as anxiety, depression, stress, loneliness, and disturbed sleep (Cho and Lee, 2017; Duke and Montag, 2017; Lin et al., 2014; Rozgonjuk et al., 2020).

Several studies have explored the role of PA as a protective factor against SA. A systematic review by Pirwani and Szabo (2024b), which synthesized evidence from 31 studies, concluded that regular PA might be a preventive measure against SA. An inverse relationship between PA and SA has also been observed in children (Raustorp et al., 2019) and across multiple adult samples (Liu et al., 2022). Exercise appears to regulate the neurobiological mechanisms of addiction, influencing central and autonomic nervous system functions (Li et al., 2020). Furthermore, regular exercisers tend to develop more utilitarian smartphone use habits. They engage with their devices for self-monitoring, goal-setting, and sports-related information-seeking rather than for hedonic activities (Pirwani and Szabo, 2024a).

Research suggests that unmet psychological needs may fuel compensatory smartphone use, with individuals relying on digital interactions to fill social and emotional gaps (Gong et al., 2023). Exercise and sports participation provide opportunities for real-life socialization, potentially reducing SA by fulfilling psychological needs through PA rather than virtual engagement. A study on smartphone use among college students found that individuals with lower PA levels had a threefold increased likelihood of smartphone abuse (Grimaldi-Puyana et al., 2020). Kim (2013) proposed that exercise rehabilitation can mitigate SA through a two-stage process: first, by fostering physical health and, subsequently, by improving psychological resilience. The present study examines differences in SA between individuals who exercise regularly and non-exercisers to investigate this relationship further.

Age and gender^1^ have been consistently identified as key predictors of SA. Csibi et al. (2019a) found that younger individuals are at higher risk for developing SA; they are the most vulnerable age group. Similarly, Guimarães et al. (2022) reported that younger individuals exhibit higher SA scores and a greater likelihood of experiencing nomophobia—the fear of being without a smartphone. A study across 41 countries confirmed that SA declines with age (Olson et al., 2023). Gender differences in SA have also been widely reported. Studies have consistently found that females exhibit higher SA scores than males (Olson et al., 2023; Wu et al., 2022; Nishida et al., 2019). Women primarily use smartphones for social networking and communication, increasing the risk of habitual or excessive use (Van Deursen et al., 2015). In contrast, men are likelier to engage in gaming and media consumption (Roberts et al., 2014). However, some studies, such as Guimarães et al. (2022), found no significant gender differences, indicating that further investigation is warranted.

Additionally, Jaalouk and Boumosleh (2018) suggested that early smartphone exposure alone does not predict long-term addiction risk; instead, long-term usage behaviors play a more significant role. While both gender and age have been implicated in the etiology of SA, their interaction with regular exercise status, defined here as at least one hour of planned exercise every week, received little attention in the literature. Although one hour of weekly exercise may be insufficient for accruing significant health benefits, it still represents a *structured and repeated effort to engage in sustained PA*, making it a reasonable definition of regular exercise. For example, a large Norwegian study concluded that “*12% of future cases of depression could have been prevented if all participants had engaged in at least 1 h of physical activity each week*.” (Harvey et al., 2018, p. 28).

Despite a growing body of literature on smartphone addiction and PA, a significant research gap remains in understanding how regular exercise interacts with SA, withdrawal symptoms, and screen time, particularly when considering demographic factors such as age and gender. While previous studies have explored the inverse relationship between PA and SA, most have not examined whether the strength of the association between screen time and addiction symptoms differs across exercisers and non-exercisers. Additionally, limited attention has been given to how gender and age influence these patterns. The present study addresses this gap by examining these variables simultaneously, offering a more nuanced understanding of smartphone-related behaviors in the context of exercise.

Therefore, the present study aims to explore the complex relationship between regular exercise and SA, examining whether exercise status is linked to SA, smartphone deprivation symptoms, gender, age, and daily screen time. Given the growing health concerns about smartphone overuse and its potential behavioral and psychological consequences, understanding how regular exercise influences these factors is crucial. To achieve this aim, the present study tested the following hypotheses:

- *H1: Regular exercise will be associated with lower levels of SA, reduced smartphone deprivation symptoms, and decreased daily smartphone screen time.*
- *H2: The relationship between SA and daily smartphone screen time will be stronger among non-exercisers than exercisers, as exercisers are likely to allocate time to PA, reducing opportunities for excessive smartphone use.*
- *H3: Consistent with past research, age will negatively correlate with SA, smartphone deprivation symptoms, and daily smartphone screen time, suggesting that younger individuals are more susceptible to problematic smartphone use.*
- *H4: In line with prior investigations, gender will be significantly associated with SA, smartphone deprivation symptoms, and daily smartphone screen time, with females reporting higher levels than males.*

By addressing these hypotheses, the present study aims to provide valuable insights into the interplay between exercise, age, gender, and smartphone-related behaviors, contributing to a better understanding of digital well-being and health-promoting behaviors.

## Methods

### Study design

This cross-sectional study examined the relationship between SA, gender, age, smartphone screen time, smartphone deprivation, and exercise status. Data were collected between November 01 and December 12, 2024, via an online survey hosted on the Qualtrics research platform, ensuring accessibility for a broad participant pool. Participants were recruited on social media, including Facebook, Instagram, and LinkedIn, and through leaflets posted at community centers across central Budapest. The volunteers were assured anonymity before providing demographic information, such as gender and age, and consent to participation in the study.

Although a formal power analysis using G*Power was not conducted prior to data collection, the final sample size (*N* = 701) exceeds the commonly recommended thresholds for multivariate analyses such as MANCOVA. According to sample size guidelines summarized by Memon et al. (2020), Roscoe (1975) suggested that a sample between 30 and 500 is adequate for most behavioral research, while Green (1991) recommended using the formula N ≥ 50 + 8 m to estimate the minimum required sample size (where *m* is the number of predictors). Based on these recommendations, the sample size used in the present study was more than sufficient to support robust group comparisons and correlation analyses across variables such as age, gender, exercise status, and smartphone use indicators.

### Ethical approval

Ethical approval for the present study was obtained from the Research Ethics Committee of the Faculty of Education and Psychology at Eötvös Loránd University (Approval No. 2024–480).

### Participants

A total of 701 participants completed the survey, with 697 valid cases included in the final analysis because four individuals did not specify their gender; therefore, they were excluded from the gender-related analyses. Participants were aged between 18 and 67 years (*M* = 23.52, SD = 7.26), including 173 males (24.8%) and 524 females (75.2%). Among them, 392 participants (56.2%) were classified as regular exercisers and 309 (44.0%) as non-exercisers, based on self-reported engagement in at least 1 h of structured PA per week.

### Measures

#### Smartphone addiction (SA)

Smartphone addiction was assessed using the Hungarian version of the Smartphone Application-Based Addiction Scale (SABAS; in Hungarian: “Rövid Okostelefon Addikció Kérdőív” [ROTAK]). This scale consists of six items rated on a 6-point Likert scale ranging from 1 (completely disagree) to 6 (completely agree). Exploratory factor analysis conducted by Csibi et al. (2019b) confirmed a one-factor structure explaining 35% of the variance, with a Cronbach’s alpha of 0.75, indicating good reliability. A cutoff score of 23 was used to classify participants as having SA. This threshold was based on the study by Peng et al. (2023), which analyzed data from over 60,000 adolescents and identified 23 as the optimal screening point. Alternative cutoff scores of 21 (Hosen et al., 2021) and 29 (Szabo et al., 2019) have been suggested in previous research. However, 23 was chosen due to its broader population validity [based on the large sample studied by Peng et al., 2023].

#### Smartphone withdrawal feelings

Smartphone deprivation was measured using the Hungarian Smartphone Withdrawal Symptom Scale (HSWSS), developed and validated by Csibi et al. (2019b). This scale assesses withdrawal-like effects when participants cannot use their smartphones for an extended period. The scale consists of nine adjectives measuring emotional and psychophysiological symptoms, including irritability, restlessness, and fatigue. Responses were recorded on a 7-point Likert scale ranging from 1 (Never) to 7 (Always). Factor analysis revealed a one-factor structure explaining 59% of the variance, and Cronbach’s alpha was 0.93, indicating excellent reliability.

#### Subjectively appraised smartphone screen time

Daily smartphone usage was assessed through self-reported average daily screen time, recorded using a slider scale ranging from 1 to 16 h. This upper limit was chosen to ensure data accuracy and prevent overestimation due to unrealistic self-reports. Given that individuals typically spend around 8 h sleeping, reporting more than 16 h of smartphone use in a day would be improbable and could indicate misreported data. Furthermore, research suggests that university students highly susceptible to SA (Csibi et al., 2019a) typically use their smartphones for anywhere between 1 and 16 h maximum per day (Ataş and Çelik, 2019), with their internet usage also reaching similar durations.

Exercise behavior was self-reported as either “Yes” or “No.” Those who indicated exercising were further asked whether they primarily engaged in team sports (“Yes” or “No”) and to report their best estimate of weekly exercise hours.

### Statistical analysis

All analyses were conducted using SPSS (v. 28) software. Descriptive statistics were calculated for SA, self-reported deprivation scores, and daily smartphone screen time (hrs). A multivariate analysis of covariance (MANCOVA) was conducted to examine the effects of gender, exercise status, and age (used as covariate) on the dependent variables. A chi-square (χ^2^) test assessed gender differences in SA prevalence based on the predefined cutoff score (i.e., 23 out of 36). Pearson product moment correlation coefficients were calculated separately for exercisers and non-exercisers to test the strength and direction of the relationships between SA, deprivation symptoms, and daily smartphone screen time.

## Results

### Sample characteristics

Of the 701 participants, 392 (56.2%) were classified as exercisers, while 309 (44.0%) were non-exercisers. The mean SA score was 19.64 (±4.90), and the mean deprivation score was 18.43 (±8.25). Daily smartphone screen time averaged 5.01 h (±2.14). The descriptive statistics separated for males and females are presented in Table 1.

**Table 1:** Descriptive statistics for smartphone addiction (SA), deprivation feelings, and screen time.

Measure	Males (Mean ± SD)	Females (Mean ± SD)	Total (Mean ± SD)
Smartphone Addiction (SA; range = 6–36)	18.79 ± 4.56	19.91 ± 4.98	19.63 ± 4.89
Smartphone deprivation feelings (range = 9–63)	17.01 ± 7.89	18.88 ± 8.34	18.41 ± 8.26
Daily smartphone screen time (hours; range = 1–16)	4.64 ± 1.85	5.12 ± 2.22	5.00 ± 2.15

### Prevalence of smartphone addiction

Using a cutoff score of 23 on the SABAS, 203 participants (29.0%) were classified at risk for SA. Chi-square tests showed no statistically significant differences in the proportion of individuals at risk for SA between the regular exercise and no-exercise groups [χ^2^(1) = 0.425, *p* = 0.514]. However, a significantly higher proportion of females (31.7%) than males (20.8%) were at risk of SA [χ^2^(1) = 7.467, *p* = 0.006].

### Effects of age on smartphone addiction, smartphone deprivation, and screen time

The MANCOVA results indicated that age was a statistically significant covariate [Pillai’s Trace = 0.051, *F*(3, 689) = 12.433, *p* < 0.001, effect site partial Eta squared (η^2^) = 0.051]. Further analysis revealed that age influenced all three outcome measures: SA scores [*F*(1, 691) = 27.313, *p* < 0.001, partial η^2^ = 0.038], deprivation symptoms [*F*(1, 691) = 24.100, *p* < 0.001, partial η^2^ = 0.034], and in average screen time [*F*(1, 691) = 12.236, *p* < 0.001, partial η^2^ = 0.017]. The correlations between age and outcome measures were negative: SA (*r* = −0.203, *p* < 0.001), deprivation feelings (*r* = −0.187, *p* < 0.001), and screen time (*r* = −0.132, *p* < 0.001), suggesting an inverse relationship between age and the outcome measures. Nevertheless, despite the statistical significance, the coefficients of determination (*r*^2^) were low (i.e., 0.041. 0.034, and 0.017, respectively), indicating that the variances accounting for these relationships were less than 5.0% in all three cases.

### Exercise status and smartphone addiction, smartphone deprivation, and screen time

The MANCOVA results showed that exercise status did not differentiate the groups in SA, deprivation symptoms, or daily smartphone screen time [Pillai’s Trace = 0.004, *F*(3,689) = 0.916, *p* = 0.433, partial η^2^ = 0.004]. Furthermore, the exercise status by gender interaction was not significant either. However, the multivariate gender main effect approached (*p* = 0.56) but did not reach the conservative level of statistical significance (*p* = 0.05). Nevertheless, based on the suggestion of Tabachnick and Fidell (2013), the present study examined the univariate effects. These tests have emerged as statistically significant in all three outcome measures. Table 2 summarizes the multivariate test results, while Table 3 presents the univariate test results.

**Table 2:** Multivariate results for gender, age, exercise status, and exercise status by gender interaction.

Effect	Wilks’ Lambda	*F*	df	*p*-value	Partial η^2^
Gender	0.989	2.533	3, 689	0.056*	0.011
Age	0.949	12.433	3, 689	< 0.001**	0.051
Exercise status	0.996	0.916	3, 689	0.433	0.004
Exercise × Gender	0.997	0.603	3, 689	0.613	0.003

*Although the multivariate effect did not reach the predefined level of statistical significance, the strong trend (*p* < 0.06) justified further examination of the univariate effects (Tabachnick and Fidell, 2013), which were statistically significant (see Table 3) for all three dependent measures. **Statistically significant.

**Table 3:** Between-subjects effects for smartphone addiction (SA), smartphone deprivation, and daily screen time, main effects and interaction.

	Dependent variable	*F*	*p*-value	Partial η^2^
Main effect: Gender	SA	3.994	0.046*	0.006
	Deprivation	4.636	0.032*	0.007
	Daily screen time	3.914	0.048*	0.006
Main effect: Exercise Status	SA	0.089	0.765	0.001
	Deprivation	0.507	0.477	0.001
	Daily screen time	2.343	0.126	0.003
Interaction: Exercise status by gender	SA	0.013	0.908	0.000
	Deprivation	1.157	0.282	0.002
	Daily screen time	0.014	0.905	0.000

* = statistically significant; SA = smartphone addiction; η^2^ = partial eta squared, effect sizes; age is the covariate.

### Differences in deprivation and screen time between high and low SA groups

Since exercise status did not make a difference in SA, deprivation symptoms, and screen time, the present study also looked at the differences in deprivation feelings and screen time between those at risk of SA (29% of the sample) and those not at risk of SA (71% of the sample). A MANCOVA, controlling age, revealed that those at risk of SA differed from the rest in these two measures [Pillai’s trace = 0.195, *F*(2, 997) = 84.32, *p* < 0.001, partial η^2^ = 0.195]. The former scored higher on both, deprivation symptom rating [means = 23.79 ± 8.47 vs. 16.24 ± 7.10; *F*(1, 698) = 137.03, *p* < 0.001, partial η^2^ = 0.164] and screen time [means = 5.88 ± 2.20 h vs. 4.65 ± 2.02 h; *F*(1, 698) = 46.76, *p* < 0.001, partial η^2^ = 0.063].

### Correlation between smartphone addiction and usage in exercisers vs. non-exercisers

A stronger correlation was observed between SA and daily smartphone screen time in non- exercisers (*r* = 0.407, *p* < 0.001) compared to exercisers (*r* = 0.274, *p* < 0.001). Fisher’s *r*-to-*z* transformation revealed that the difference between the correlation coefficients was statistically significant (*z* = 1.97, *p* < 0.05), suggesting a more substantial SA-screen time relationship in non-exercisers compared to exercisers. Additionally, smartphone deprivation was significantly correlated with SA in both groups (exercisers: *r* = 0.529, *p* < 0.001; non-exercisers: *r* = 0.572, *p* < 0.001), but the correlations were not statistically significantly different. Similarly, smartphone deprivation correlated positively with daily smartphone screen time, with values slightly lower in exercisers (exercisers: *r* = 0.178, *p* < 0.001; non-exercisers: *r* = 0.251, *p* < 0.001), but again, the correlation coefficients did not differ statistically significantly from one another. Finally, the self-reported weekly hours of exercise did not correlate statistically significantly with SA, smartphone deprivation feelings, or screen time.

## Discussion

The present study examined the relationship between SA, deprivation symptoms, daily smartphone screen time, exercise status, gender, and age. Results indicated that exercise status did not significantly influence SA, deprivation, or screen time. However, non-exercisers showed a stronger association between SA and screen time, suggesting they may engage with smartphones more problematically than exercisers. This aligns with recent findings (Pirwani and Szabo, 2024a) that suggest sports and exercise-related smartphone use may counterbalance hedonic use.

### Originality and significance

This study provides a novel contribution to the literature by analyzing the strength of association between smartphone addiction and screen time separately in exercisers and non-exercisers. The finding that this association is significantly weaker in exercisers is original and suggests a qualitative difference in smartphone engagement, potentially reflecting more utilitarian rather than hedonic usage patterns among those who engage in PA. To our knowledge, this pattern of association has not been previously examined in a single, large-sample study. These findings are significant as they support the notion that exercise may not directly reduce smartphone addiction scores but could relate to less problematic smartphone use behaviors. This implies that exercise may influence the quality rather than the quantity of smartphone engagement, offering a more nuanced perspective that moves beyond screen time as a sole indicator of risk.

Contrary to the present study’s findings, systematic reviews suggest that exercise can mitigate SA (Azam et al., 2020; Pirwani and Szabo, 2024b), potentially because PA is more effective as an intervention than a protective factor. While exercisers did not show a lower prevalence of SA risk, their weaker SA-screen time link suggests that smartphone use may be less problematic. This may stem from their tendency to use smartphones for health and fitness tracking rather than for hedonic purposes (Pirwani and Szabo, 2024a). The increasing integration of smartwatches and fitness apps likely contributes to higher screen time without fostering addictive behaviors.

Despite a seemingly greater addictive tendency demonstrated by the stronger SA-screen time relationship in non-exercisers compared to exercisers, the former group did not experience lesser feelings of deprivation than exercisers, nor was the strength of the correlations between SA and deprivation symptoms different in the two groups. The magnitude of these correlations was medium, which could result from a wide range of associations between these measures. Some individuals may experience painful feelings of deprivation or even cravings when they are prevented from accessing their smartphones, while others may feel minimal discomfort. In contrast, the relationship between smartphone deprivation and screen time was small in both groups, accounting for less than 10% of the variance.

While it was reported earlier (Eide et al., 2018) that smartphone deprivation could cause withdrawal symptoms, the strength of the association between screen time and the intensity of deprivation symptoms has not been evaluated. Based on the current results, this relationship is weak. The finding could be associated with utilitarian use (i.e., work or study) and hedonic use (i.e., games, social media), with the latter being linked to SA (Vujić and Szabo, 2022). Indeed, obstructed access in utilitarian use may cause anger, irritation, or akin affective states, but this surmise is subject to further research. At the same time, high SA may be associated with more screen time and stronger subjective feelings of deprivation, as demonstrated in the current study. Therefore, regardless of exercise status, adults at risk of SA report more smartphone screen time along with more intense feelings of deprivation when smartphone access is not possible.

Regrettably, the present study did not assess the purpose of smartphone use. Previous studies found that sports-related smartphone use can counteract the effects of hedonic use (Pirwani and Szabo, 2024a). Furthermore, in contrast to the present study’s findings, Ergun and Guzel (2018) found a positive association between greater exercise participation and increased smartphone use. Given that SA is linked to hedonic use rather than overall screen time (Vujić and Szabo, 2022), future research should investigate whether the type of smartphone use mediates the relationship between PA and SA.

Results confirmed significant gender differences in SA, smartphone deprivation, and screen time, with females exhibiting higher values across all outcome measures. However, the small effect sizes suggest that while these differences are statistically significant, their practical significance may be limited. On the one hand, these findings are consistent with previous research indicating that women report higher levels of SA than men across various age groups, including adolescents (Kalaitzaki et al., 2022; Yavuz et al., 2019). On the other hand, the small effect sizes align with studies that found no significant gender differences (Gao et al., 2023). However, they do not support findings suggesting that males exhibit stronger associations with SA (Wang et al., 2019). These inconsistencies may stem from cultural or methodological differences in the assessment of SA. Some studies assess overall SA, while others focus on specific subtypes, such as mobile gaming addiction, which may be more prevalent among men. Future research should distinguish between different forms of smartphone engagement (e.g., social media use vs. gaming) to better understand gender differences in SA.

The present study also found that younger participants exhibited significantly higher SA, deprivation symptoms, and daily usage, consistent with prior research (Al-Balhan et al., 2018). Age has long been recognized as a strong predictor of SA, with similar findings reported in Iran (Soleymani et al., 2019), Spain (León-Mejía et al., 2020), and Portugal (Dias et al., 2019). However, in the present study, similarly to gender effects, age was weakly correlated with SA, feelings of deprivation, and screen time. Indeed, the shared variance of these variables with age was less than 5%.

This finding may be due to a categorical rather than continuous relationship between age and SA (Csibi et al., 2019a). For example, younger individuals in a specific age-group category may experience a greater fear of being without their phones (nomophobia), making them more susceptible to deprivation symptoms and more compulsive use. Andone et al. (2016) suggested that hedonic smartphone use is negatively correlated with age, meaning younger users are more likely to engage in hedonic activities, which increases addiction risk (Bae, 2017; Vujić and Szabo, 2022). As hedonic use is a key predictor of addiction, younger individuals may benefit from interventions focusing on self-regulation and mindful smartphone use.

### Strengths

The present study’s strengths include its large sample size, use of validated psychometric tools (SABAS and HSWSS), and robust statistical methods. The comparison of exercisers and non-exercisers in relation to addiction-related variables provides valuable insights into different patterns of smartphone use. While the data were collected only in Hungary, the study’s focus on behavioral trends and demographic characteristics still offers relevant contributions to the field of digital health and addiction.

### Limitations

Despite its strengths, the present study has several limitations. Self-report measures could introduce bias, as participants may overestimate or underestimate smartphone screen time and exercise duration due to memory bias or social desirability. The voluntary, anonymous online research limits control over participant characteristics, affecting sample representativeness and response reliability. The gender imbalance (only 25% males) may skew findings, as results could reflect female smartphone usage patterns more than male ones. Furthermore, a minimum of 1 h of weekly exercise, satisfying the eligibility for participation in the present study, may have buffered the strengths of the relationship between regular exercise and SA. The study’s focus on only Hungarian participants limits generalizability, as cultural differences in smartphone use may influence results. Finally, the cross-sectional design prevents causal conclusions, requiring longitudinal studies to explore whether age, gender, or exercise directly impact SA.

### Future directions

Future research should investigate intervention-based approaches along with objective exercise and smartphone use measures to determine whether specific types of exercise can reduce SA. Distinguishing between hedonic and utilitarian smartphone use could also provide insights into how different engagement patterns contribute to addiction risk. Understanding these nuances may help design more effective strategies for promoting healthier smartphone use, particularly among those vulnerable to dependence.

## Conclusion

In the present study, regular exercise status was unrelated to SA, subjectively reported smartphone deprivation symptoms, or self-reported daily screen time. However, gender effects have emerged because females exhibited higher SA, more intense deprivation symptoms, and more screen time than males. Nevertheless, the effect sizes were small, which calls for the cautious interpretation of the gender-related differences. Similarly, regardless of exercise status, age was weakly correlated with SA, feelings of deprivation, and daily screen time. However, the shared variance was less than 5%. Future research should explore the nuanced interactions between regular exercise, gender, and age on SA by adopting longitudinal designs and objective smartphone usage data to clarify causal relationships and better capture individual differences in behavioral patterns.

## Data Availability

The datasets presented in this study can be found in online repositories. The names of the repository/repositories and accession number(s) can be found here: https://data.mendeley.com/datasets/9xs4gwd8wc/1.

## Footnotes

^1^In the present study, “gender” is used instead of biological sex, recognizing that social factors and personal values shape gender. Given the phrasing of the question (“What is your gender?”), it is assumed that participants responded based on their self-identified gender identity.
